# Pharmacokinetics and outcome of high-dose melphalan followed by autologous stem cell transplantation in dialysis-dependent patients with multiple myeloma

**DOI:** 10.1016/j.lrr.2025.100522

**Published:** 2025-06-19

**Authors:** Rabea Mecklenbrauck, Bernhard M.W. Schmidt, Heike Bähre, Annamaria Brioli, Arnold Ganser, Florian H. Heidel, Felicitas Thol

**Affiliations:** a Department of Hematology, Hemostasis, Oncology and Stem Cell Transplantation, Hannover Medical School, Hannover, Germany; b Department of Nephrology and Hypertension, Hannover Medical School, Hannover, Germany; c ZFA Metabolomics, Hannover Medical School, Hannover, Germany; d Cellular Therapy Center (CTC), Hannover Medical School, Hannover, Germany

**Keywords:** Multiple myeloma, Autologous stem cell transplantation, High-dose melphalan, Hemodialysis

## Abstract

High-dose melphalan followed by autologous stem cell transplantation (ASCT) remains the standard of care for fit patients with multiple myeloma (MM). However, individuals who are dependent on hemodialysis are frequently excluded from ASCT. Recommendations on chemotherapy dosing and hemodialysis scheduling vary in literature and definite conclusions are impeded by the heterogeneity of cohorts.

We aimed to evaluate the safety and efficacy of ASCT in patients with MM and end-stage renal disease and to examine the pharmacokinetics of melphalan on a fixed schedule of melphalan infusion and hemodialysis.

The outcome of 13 patients undergoing ASCT while being on hemodialysis between 2000 and 2022 was retrospectively analysed and compared to matched hemodialysis-independent patients. Melphalan plasma concentrations were measured in 4 hemodialysis-dependent and 5 independent patients.

Plasma concentrations of hemodialysis-dependent patients were comparable to hemodialysis-independent patients with a 6-hour interval between melphalan infusion and hemodialysis (*p* = 0.9). The rate of immediate side effects of high-dose melphalan was significantly higher in 13 dialysis-dependent patients compared to 47 matched controls despite not having prolonged neutropenia (*p* = 0.9). Overall survival both from d_0_ of ASCT and diagnosis was comparable (*p* = 0.33 and *p* = 0.17, respectively).

Thus, adopting the proposed schedule and management of immediate side effects make ASCT a safe option for myeloma patients with end-stage renal disease.

## Highlights

• Dialysis-dependent myeloma patients experience more toxicities at transplantation.
• Long-term outcome is comparable to patients independent of dialysis.
• Fixed schedule of melphalan and dialysis mimics pharmacokinetics without dialysis.
• High-dose melphalan should be considered for dialysis-dependent myeloma patients.

## 1 Introduction

Autologous stem cell transplantation (ASCT) has long been recognized as an important treatment modality for fit newly diagnosed as well as relapsed patients with multiple myeloma (MM) [1,2]. Even in the era of novel and highly effective combination therapies ASCT leads to a significant prolongation of progression-free survival (PFS) and remains standard of care for fit patients [3]. Interestingly, most studies could not show a benefit for overall survival (OS) when ASCT was compared to triplet combination therapy without ASCT [3,4]. Furthermore, due to toxicity associated with ASCT a careful risk assessment is mandatory prior to planning ASCT.

At diagnosis 20–30 % of patients with MM present with renal impairment while about 50 % will develop renal impairment over the course of the disease [5].

Reduced renal function is associated with shorter OS and, notably, those patients being dependent on hemodialysis have an especially poor outcome[6]. Therefore, the risk of ASCT is frequently debated in MM patients being dependent on hemodialysis. Here, inpatient mortality is increased although recent advances in supportive care seem to improve the immediate risks associated with ASCT [7]. Interestingly, long-term outcome reported in more recent studies appeared to be similar to patients without renal impairment [8].

To date, there is no consistent consensus on the dosing of melphalan in hemodialysis-dependent patients and how the infusion of melphalan and stem cells should be scheduled in regard to hemodialysis.

Early studies on the pharmacokinetics of melphalan have found that renal impairment does not seem to have an influence on melphalan clearance; there was in fact a higher inter-patient variability regardless of renal function but no negative impact of a reduced eGFR [9]. Nevertheless, a dose-reduction of melphalan is recommended depending on eGFR. The dose which is most commonly given to patients with renal impairment in several retrospective studies is 140mg/m^2^ melphalan over two days [10,11], however, applied doses described in literature vary between 70 and 200mg/m^2^ [12,13]. Even more inconsistencies are reported regarding the sequencing of melphalan application and hemodialysis. It is not entirely clear whether melphalan is removed by hemodialysis as melphalan half-life in water is short which could interfere with measurements in the dialyzed fluids. Toxicity of 200mg/m^2^ melphalan was higher compared to reduced doses in patients with renal impairment and, strikingly, a dose reduction did not confer a disadvantage in survival [14].

In the present study, we report on our experience with hemodialysis-dependent patients undergoing ASCT and propose a fixed schedule of melphalan application and hemodialysis which mimics the pharmacokinetics of hemodialysis-independent patients.

## 2 Materials and methods

### 2.1 Patient cohort and statistical analysis

For the analysis of adverse events and outcome, we report on 13 patients requiring hemodialysis who received high-dose melphalan followed by ASCT at our center between 2000 and 2022. As a control we analysed 481 ASCTs that were performed on 331 different patients who were independent of hemodialysis.

We matched dialysis-independent patients with dialysis-dependent patients in a 4:1 ratio using propensity score matching and the nearest neighbor method. After matching we excluded patients which were matched more than once so that 47 dialysis-independent patients were matched to 13 patients on dialysis.

Side effects were monitored according to the CTCAE definitions. Neutrophilic engraftment was defined as the first of at least 3 consecutive days with >500 neutrophilic granulocytes/µl. Platelet engraftment was defined as the first of three consecutive days with >20×10^9^ platelets/L without transfusions.

OS was measured from both diagnosis and d_0_ of ASCT until day of death for any cause. Patients lost to follow up were censored on the last day of follow up.

All statistical analyses were performed using R (version 4.3.2, packages cobalt, cowplot, ggsurvplot, matchit, smplot2, survival, survivalAnalysis, survminer). To compare survival the Kaplan-Meier method with log-rank test and Cox proportional hazard models were used. Categorical variables were compared using the χ ^2^ test and continuous variables were compared using the Wilcoxon rank-sum test.

### 2.2 Measurements

For the study of pharmacokinetics, we collected plasma samples of 4 patients who received melphalan while being hemodialysis-dependent. One of these patients had plasma cell leukemia and was henceforth considered for analysis regarding melphalan concentrations but not included in the outcome analysis. Melphalan concentrations were compared to those of 5 patients who were not hemodialysis-dependent at ASCT. The renal function of three of those patients was classified as KDIGO G2A1, of another patient as G3aA1 and one patient had normal renal function. The four hemodialysis-dependent patients received standard dialysis treatment using a high-flux dialyzer for 2 to 4 h exactly 6 h after the start of the melphalan infusion. Melphalan was given over 30 min on days 1 and 2. Stem cells were infused at least 48 h after the second melphalan infusion. Each patient received at least 2 × 10^6^ CD34+ cells/kg body weight. For 3/4 hemodialysis-dependent patients melphalan was dosed at 140mg/m^2^ and at 200mg/m^2^ for 1/4, whereas for hemodialysis-independent patients melphalan was given at 200mg/m^2^ (Supplemental Table S1).

Melphalan concentrations in plasma were measured at the following time points:

- Baseline before melphalan infusion day 1
- Directly after melphalan infusion day 1
- Before hemodialysis day 1 (dialysis patients only)
- After hemodialysis day 1 (dialysis patients only)
- Baseline before melphalan infusion day 2
- Directly after melphalan infusion day 2
- Before hemodialysis day 2 (dialysis patients only)
- After hemodialysis day 2 (dialysis patients only)
- Trough level 20 h after last melphalan infusion

The samples were centrifuged at 200 g for 10 min and plasma was stored at −80 °C until the day of measurement. Melphalan concentrations were measured by mass spectrometry.

As a control and to establish the method we used pure melphalan (melphalan powder, Sigma Aldrich, Taufkirchen). Plasma samples taken before the application of the first dose of melphalan served as the negative control.

## 3 Ethical approval

The study was approved by the local ethics committee (No. 9605_BO_S_2021). Informed consent was obtained from all patients prior to sample collection.

## 4 Results

### 4.1 Characteristics and outcome of hemodialysis-dependent in comparison to hemodialysis-independent patients

Thirteen ASCTs were performed at our centre between 2000 and 2022 while the patients were dependent on hemodialysis. One of these patients was on hemodialysis only for the time of transplantation and was successfully weaned of hemodialysis directly after ASCT. Seven of these 13 patients (54 %) had λ light chain (LC), five (38 %) IgG κ and one (8 %) IgG λ as myeloma protein. Nine patients (69 %) were diagnosed at stage IIIB according to Durie-Salmon, six (46 %) were classified as International Staging System (ISS) stage III.

We matched these 13 patients to hemodialysis-independent patients undergoing ASCT in the same time period (*n* = 47). Patients were matched for sex, age at transplantation and transplantation as first line therapy or in relapse. After matching there was no significant difference regarding sex (*p* = 1), age (median 60 and 59.72 years respectively, *p* = 0.693) and first ASCT vs. ASCT at relapse (*p* = 1) (Table 1). As expected, patients being on hemodialysis at the time of transplantation tended to have more advanced disease according to ISS already at diagnosis: 46 % in the hemodialysis-dependent cohort presented with ISS III in comparison to only 6 % in the non-hemodialysis cohort (*p* = 0.003). Interestingly, no difference in performance status (*p* = 0.311) and time from diagnosis to transplantation (*p* = 0.207) was observed. However, a median of two lines of therapy in hemodialysis patients was higher than in non-hemodialysis patients who on average received only one line of therapy before ASCT (*p* = 0.005). The median dose of melphalan was 200 mg/m^2^ (range 140 - 200 mg/m^2^) in non-hemodialysis patients and 140 mg/m^2^ (range 100 - 200 mg/m^2^) in hemodialysis patients (Table 1).

**Table 1:** Baseline characteristics of hemodialysis-dependent patients in comparison to matched hemodialysis-independent patients.

	HD-dependent patients (*n* = 13)	HD-independent patients (*n* = 47)	p
**Age at transplantation**			0.693
**Median** – **years**	60	59.72	
**Range** – **years**	33 – 69	30.89 – 74.39	
**Patient sex**			1
**Male – n ( %)**	8 (62)	28 (60)	
**Female – n ( %)**	5 (38)	19 (40)	
**ECOG performance status at diagnosis**			0.311
**ECOG 0–1 – n ( %)**	12 (92)	38 (81)	
**ECOG ≥2 – n ( %)**	1(8)	2 (4)	
**No information – n ( %)**	0	7 (15)	
**ASCT**			1
**In first line – n ( %)**	9 (69)	34 (72)	
**After relapse – n ( %)**	4 (31)	13 (28)	
**ISS stage**			
**I – n ( %)**	0	7 (15)	0.003
**II – n ( %)**	1 (8)	3 (6)	
**III – n ( %)**	6 (46)	3 (6)	
**No information – n ( %)**	6 (46)	34 (72)	
**Lines of therapies before ASCT**			0.005
**Median**	2	1	
**Range**	1 – 5	1 – 4	
**No information – n ( %)**	0	6	
**Time from diagnosis to ASCT**			0.207
**Median – days**	234	374	
**Range – days**	172 – 1724	128 – 3887	
**No information – n ( %)**	1 (8)	0	
**Dose Melphalan**			<0.001
**Median – mg/m^2^**	140	200	
**Range – mg/m^2^**	100 – 200	140 – 200	
**No information – n ( %)**	0	7 (15)	

Abbreviations: ASCT, autologous stem cell transplantation; ECOG: Eastern Cooperative Oncology Group; HD, hemodialysis.

Patients on hemodialysis had a higher rate of adverse events while undergoing ASCT. All 13 dialysis-dependent patients experienced adverse events while undergoing ASCT. Both the incidence of diarrhea, neutropenic fever and mucositis (≥ grade 3) were significantly higher in patients being dependent on hemodialysis (Table 2). All hemodialysis-patients developed neutropenic fever while only 43 % of the hemodialysis-independent patients were affected (*p* = 0.003) (Table 2). Mucositis was more prevalent in hemodialysis-dependent at 85 % in comparison to 40 % of the non-hemodialysis patients (*p* = 0.043). Regarding therapy-associated diarrhea two-thirds of hemodialysis-dependent patients were classified as having ≥ grade 3 compared to only 6 % in the control group (*p* < 0.001).

**Table 2:** Side effects of melphalan treatment and ASCT.

	HD-dependent patients (*n* = 13)	HD-independent patients (*n* = 47)	p
**Diarrhea**			<0.001
**< Grade 3 – n ( %)**	4 (31)	34 (72)	
**≥ Grade 3 – n ( %)**	9 (69)	3 (6)	
**No information – n ( %)**	0	10 (21)	
**Mucositis**			0.043
**< Grade 3 – n ( %)**	2 (15)	21 (45)	
**≥ Grade 3 – n ( %)**	11(85)	19 (40)	
**No information – n ( %)**	0	7 (15)	
**Febrile neutropenia**			0.003
**< Grade 3 – n ( %)**	0	21 (45)	
**≥ Grade 3 – n ( %)**	13 (100)	20 (43)	
**No information – n ( %)**	0	6 (13)	
**Days to regeneration of neutrophiles**			0.943
**Median – days**	13.5	14	
**Range – days**	10 **–** 17	9 **–** 20	
**No information – n ( %)**	1 (8)	11 (23)	
**Days to regeneration of thrombocytes**			0.03
**Median – days**	11	10	
**Range – days**	9 - 12	8 - 13	
**No information – n ( %)**	2 (15)	4 (9)	

Median time to neutrophil regeneration was 13.5 days (range 10 – 17 days) in hemodialysis-dependent vs. 14 days (range 9 – 20 days) in hemodialysis-independent patients (*p* = 0.943) (Table 2). While the regeneration of platelet counts was statistically significantly longer in hemodialysis patients, the numerical difference was small (median 11 vs. 10 days, *p* = 0.03). Therefore, this difference appears not to be clinically relevant.

There was a trend for higher mortality at 60 and 100 days after ASCT for hemodialysis patients (60-day mortality: HR 3.80, 95 %CI 0.95 – 15.2, *p* = 0.059; 100-day mortality: HR 3.11, 95 %CI 0.83 – 11.6, *p* = 0.091) which may be caused by the higher incidence of toxicities.

**Fig. 1:**
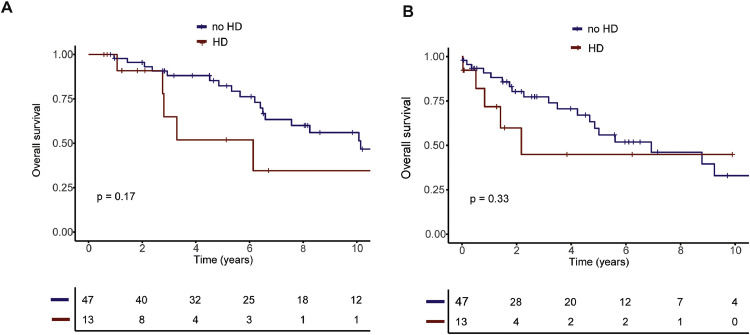
Overall survival of hemodialysis-dependent patients in comparison to matched hemodialysis-independent patients. (A) OS calculated from diagnosis and (B) calculated from d_0_ of ASCT. *Abbreviations: HD, hemodialysis.*

We then compared OS between hemodialysis-patients and the matched non-hemodialysis patients undergoing ASCT for exploratory purposes. Here, we only saw a slightly shorter OS for the hemodialysis-dependent patients which did not reach significance (HR 1.99, 95 %CI 0.73 – 5.39, *p* = 0.17) (Fig. 1A). Importantly, OS calculated from the date of ASCT was also not different (HR 1.63, HR 0.60 – 4.39, *p* = 0.33) (Fig. 1B).

### 4.2 Pharmacokinetics of melphalan in hemodialysis-dependent patients

**Fig. 2:**
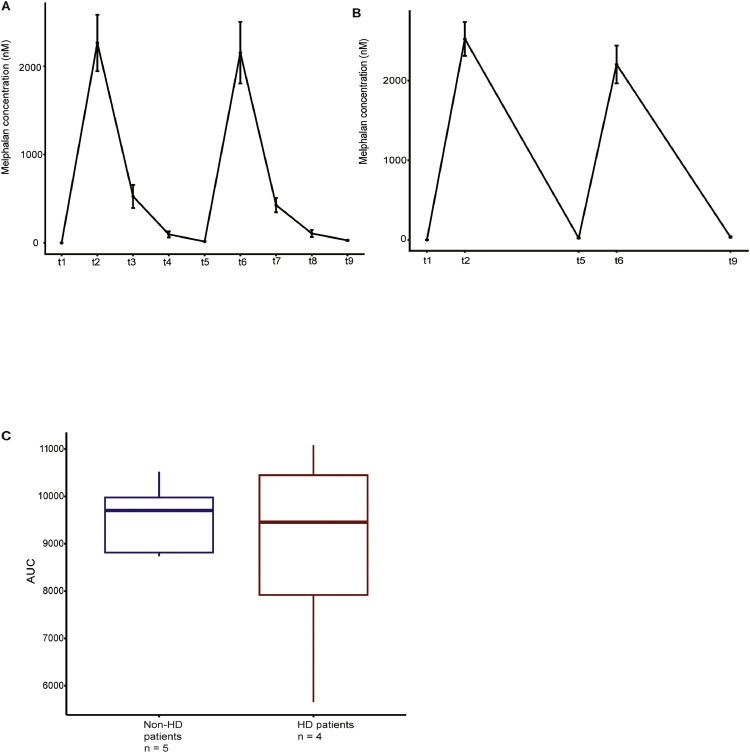
Melphalan plasma concentrations of hemodialysis-patients compared with dialysis-independent patients. (A) Line graph representing the mean melphalan concentration and standard deviation at each given time point for hemodialysis-dependent patients. *t1 = before melphalan day 1, t2 = after melphalan day1, t3 = before dialysis day 2, t4 = after dialysis day 2, t5 = before melphalan day 2, t6 = after melphalan day 2, t7 = before dialysis day 2, t8 = after dialysis day 2, t9 = trough level 20 h after melphalan*. (B) Line graph representing the mean melphalan concentration and standard deviation at each given time point for non-hemodialysis patients, *t1 = before melphalan day 1, t2 = after melphalan day1, t5 = before melphalan day 2, t6 = after melphalan day 2, t9 = trough level 20* h *after melphalan,* (C) Boxplot comparing the AUC of hemodialysis- and non-hemodialysis patients *Abbreviations: HD, hemodialysis; AUC, area under curve.*

To date there is no standardized protocol of how to sequence the infusion of melphalan and hemodialysis. We established a schedule of hemodialysis for 2 to 4 h, starting exactly 6 h after melphalan infusion on both days after melphalan infusion. In order to examine the exposure to melphalan with this protocol, in addition to the retrospective analysis, we prospectively measured the plasma concentrations of melphalan at nine different time points in three patients with multiple myeloma on hemodialysis and one patient with plasma cell leukemia who received either 140mg/m^2^ or 200mg/m^2^ melphalan. The plasma concentrations were compared to five patients independent of dialysis. These patients received 200mg/m^2^ melphalan. Plasma concentrations for each group can be seen in Fig. 2. Comparing the area under curve (AUC) we found no significant difference (*p* = 0.9) between patients on hemodialysis and hemodialysis-independent patients.

## 5 Discussion

Renal impairment is a common complication of MM; however, data on hemodialysis patients with MM are scarce as these patients are typically excluded from clinical trials.

In this study, we report on the experience with hemodialysis-dependent MM patients undergoing ASCT in our center.

Here, 3 % of all patients undergoing ASCT were hemodialysis-dependent, in accordance with published evidence which reports an overall rate of long-term dialysis-dependence in MM patients of approximately 5 %. Although ASCT prolongs PFS and hemodialysis is not an exclusion criterion for ASCT eligibility, there are no clearly established treatment guidelines for hemodialysis-dependent patients, especially regarding dosing of melphalan and timing of hemodialysis. In this subset of patients, ASCT might be considered too risky for two main reasons: (A) patients who become hemodialysis-dependent because of MM may have more comorbidities, (B) ASCT in hemodialysis-dependent patients does not only require hematological but also nephrological expertise and a specialized dialysis unit on site.

To our knowledge, our results are the first report of a comparison between pharmacokinetics of melphalan plasma concentration from hemodialysis- and non-hemodialysis patients while on a fixed schedule of hemodialysis. With our regimen of melphalan 140mg/m^2^ over two days and hemodialysis starting exactly 6 h after melphalan infusion in hemodialysis-dependent patients, we mimic pharmacokinetics of hemodialysis-independent patients treated with melphalan 200mg/m^2^ over two days. The AUC with this regimen is comparable showing that we reach a similar exposure to melphalan.

In our cohort, the risk of adverse events associated with hemodialysis-dependence during ASCT is significantly higher than in the matched control group. Specifically, we observed a higher rate of diarrhea, mucositis and febrile neutropenia. This is in line with previously published analyses reporting on a higher rate of toxicities[12].

However, there was no difference in recovery of neutrophils. Therefore, the difference in side effects is unlikely to be related to a delayed hematological recovery in hemodialysis-dependent patients.

Considering that we achieve similar pharmacokinetics of melphalan (including AUC) with our fixed regimen (dosing of melphalan and timing of hemodialysis) in our group of hemodialysis-dependent patients as compared to hemodialysis-independent patients, it might appear surprising that we observed more adverse events in hemodialysis-dependent patients.

This might be explained by the fact that melphalan is mainly bound to albumin. Patients with renal insufficiency tend to have lower plasma albumin concentrations which may result in higher concentrations of unbound melphalan[15]. However, other factors, independent of melphalan dosing, may explain the increased rate of adverse effects. In our cohort, hemodialysis-dependent patients had more advanced disease stages. Additionally, this patient cohort might also have other comorbidities, which may explain the higher grades of gastrointestinal toxicities. Thus, the higher rate of immediate toxicities might reflect a more fragile and comorbid patient population.

OS was similar in the group of hemodialysis-dependent patients as compared to non-hemodialysis-dependent patients undergoing ASCT both from the timepoint of diagnosis and from ASCT. Due to the intrinsic limitations of the study, such as the small patient size, the retrospective nature and the different disease stage of the two populations, a formal comparison of the survival of hemodialysis vs. non-hemodialysis patients is not possible and the data should be considered only as descriptive. Nevertheless, the analysis clearly shows that ASCT is a feasible option for hemodialysis-dependent patients. An effective management of relevant side effects is of pivotal importance in this patient subset, due to the higher risk of gastrointestinal side effects and infectious complications.

The fixed interval between melphalan infusion and hemodialysis established in our center mimics the plasma concentrations of hemodialysis-independent patients and may be more widely adopted. Standardized protocols for ASCT in dialysis-dependent patients should be established to allow these patients to have access to this treatment modality.

## CRediT authorship contribution statement

**Rabea Mecklenbrauck:** Writing – review & editing, Writing – original draft, Investigation, Formal analysis, Data curation, Conceptualization. **Bernhard M.W. Schmidt:** Writing – review & editing, Formal analysis, Data curation. **Heike Bähre:** Writing – review & editing, Data curation. **Annamaria Brioli:** Writing – review & editing, Writing – original draft, Formal analysis, Data curation. **Arnold Ganser:** Writing – review & editing, Data curation. **Florian H. Heidel:** Writing – review & editing, Data curation. **Felicitas Thol:** Writing – review & editing, Writing – original draft, Investigation, Formal analysis, Data curation, Conceptualization.

## Declaration of competing interest

BMWS received lecture fees and honoraria from ADVITOS, Amgen, Bayer Vital, Berlin Chemie-Menarini, CytoSorbents, Daichii Sankyo, Miltenyi, Pocard, not related to this article. AB has participated in advisory boards from BMS, Janssen, GSK, Takeda and Sanofi and received honoraria or travel support from BMS, Janssen, GSK, Sanofi, Amgen and AstraZeneca. FHH served as an advisor for Novartis, CTI, Celgene/BMS, Janssen, Abbvie, GSK, Merck and AOP and received research funding from Novartis, Celgene/BMS and CTI, not related to this article.

## Data Availability

Individual patient data will not be made available in order to maintain health information privacy. De-identified melphalan concentration measurements will be shared upon reasonable request to the corresponding author.

## Appendix Supplementary materials

Image, application 1
