# Supplementary material for: Pharmacokinetics and outcome of high-dose melphalan followed by autologous stem cell transplantation in dialysis-dependent patients with multiple myeloma
Source: Leuk Res Rep. 2025 Jun 19;24:100522. doi: 10.1016/j.lrr.2025.100522 (PMC12264629; doi:10.1016/j.lrr.2025.100522)
Supplement: Supplementary file 1 [file mmc1.docx]

| **Patient** | **Mode of dialysis** | **Machine** | **Maximal blood flow (ml)** | **Duration of dialysis (hours)** |
| --- | --- | --- | --- | --- |
| 1 | HDF | Polyflux 170H | 300 | 4 |
| 2 | HD | Polyflux 170H | 200 | 2^1^ |
| 3 | HD | Polyflux 170H | 220 | 4 |
| 4 | HDF | Polyflux 170H | 180 | 3^2^ |

**Supplemental Table S1: Overview of hemodialysis in the 4 patients in the pharmacokinetic cohort**

**^1^** duration reduced because patient was dialysed for the first time

^2^ duration reduced because of hypotonia

Abbreviations: HD, hemodialysis; HDF, hemodiafiltration
